# Habitual Breakfast Patterns Do Not Influence Appetite and Satiety Responses in Normal vs. High-Protein Breakfasts in Overweight Adolescent Girls

**DOI:** 10.3390/nu11061223

**Published:** 2019-05-29

**Authors:** Steve M. Douglas, Adam W. Byers, Heather J. Leidy

**Affiliations:** Department of Nutrition Science, Purdue University, 700 W State Street, West Lafayette, IN 47907, USA; dougla53@purdue.edu (S.M.D.); byers11@purdue.edu (A.W.B.)

**Keywords:** breakfast, protein, habitual breakfast patterns, fullness, intake

## Abstract

Differences in postprandial insulin, glucose, and/or free fatty acid concentrations, following the consumption of breakfast, have been demonstrated to be dependent on habitual breakfast patterns. This study examined the effects of habitual breakfast patterns on postprandial appetite, satiety, and hormonal responses along with daily food intake following the consumption of normal-protein (NP) vs. higher-protein (HP) breakfasts in overweight adolescents. Thirty-seven girls (age: 19 ± 1 year; BMI: 29.0 ± 3.4 kg/m^2^) participated in the semi-randomized crossover design study. Participants were grouped according to whether they habitually skipped (SKIP, *n* = 18) or consumed breakfast (CONSUME, *n* = 19), and consumed a NP (350 kcal; 13 g protein) or HP (350 kcal; 35 g protein) breakfast for 3 days/pattern. On day 4, breakfast was provided, and appetite questionnaires and blood samples were collected throughout an 8 h testing day. Daily food intake was also assessed. Regardless of habitual breakfast patterns, the consumption of HP breakfast led to greater daily fullness (29,030 ± 6,010 min × mm) vs. NP breakfast (26,910 ± 5580 min × mm; *p* = 0.03). Daily protein consumption was greater (98 ± 15 g vs. 78 ± 15 g), and carbohydrate consumption was lower (331 ± 98 g vs. 367 ± 94 g) with HP vs. NP (both, *p* < 0.001). No other differences were observed. These data suggest that the recommendation to consume a HP breakfast for improved satiety and ingestive behavior is appropriate for overweight adolescent girls, regardless of habitual breakfast patterns.

## 1. Introduction

Childhood and adolescent overweight/obesity remains a global public health crisis. Although the prevalence of obesity in younger children is plateauing, the prevalence of obesity in adolescents continues to increase, with current trends at 20.6% [1,2]. In identifying potential behavioral strategies for improvements in obesity-related outcomes in this age group, the daily consumption of breakfast has become one nutrition intervention of interest. This is especially relevant in adolescent girls since they exhibit the greatest frequency of skipping breakfast [3,4].

As discussed in several recent reviews [5,6], the consumption of breakfast elicits acute improvements in appetite, satiety, and glycemic control compared to skipping the morning meal. However, these responses are more robust when the meals contain higher-protein foods compared to lower-protein foods [7,8]. In addition, long-term data from our lab also demonstrated chronic improvements in glycemic control, reductions in daily food intake, and the prevention of unhealthy body fat gain following the habitual consumption of higher-protein breakfasts compared to skipping breakfast or consuming a normal-protein breakfast [9,10]. However, a number of investigators have proposed that habitual breakfast patterns influence ingestive behavior, metabolic-, and weight management outcomes. 

Schlundt et al. examined the effects of breakfast consumption compared to breakfast skipping and stratified based on habitual breakfast patterns [11]. The change in habitual dietary habits (i.e., habitual breakfast consumers beginning to skip breakfast or habitual breakfast skippers beginning to consume breakfast) tended to elicit the greatest effect on weight loss [11]. Along these lines, Thomas et al. [12] and Alwattar et al. [8] reported differences in postprandial insulin, glucose, and/or free fatty acid concentrations that were dependent on habitual breakfast patterns. Lastly, Long et al. reported lower postprandial satiety following a higher-protein test meal in those who habitually ate a higher-protein diet compared with those who did not [13]. Collectively, these studies support the continued examination of habitual eating habits on obesity-related outcomes. 

Thus, the current study sought to extend the existing evidence to examine the effects of habitual breakfast patterns and breakfast composition on postprandial appetite, satiety, and hormonal responses along with daily food intake in overweight adolescent girls. We hypothesized that the response to breakfast will be more robust in habitual breakfast skippers than consumers, and that the consumption of a higher-protein breakfast (HP) will facilitate greater changes in the aforementioned mechanisms of appetite regulation, and subsequent changes in perceived appetite when compared to a normal-protein breakfast (NP). 

## 2. Materials and Methods

### 2.1. Experimental Design

Thirty-seven overweight and obese adolescent girls participated in the following randomized crossover design breakfast study. The participants were grouped according to habitual breakfast patterns. Participants were asked to randomly consume either a NP breakfast meal at home for 3 days or a HP breakfast meal at home for 3 days. On day 4 of each pattern, the participants came to our facilities in the morning to complete the respective 8 h testing day. The respective breakfast was consumed. Pre- and post-breakfast appetite, satiety-related blood samples, and questionnaires were completed every 30 min throughout the 8 h day. At the end of the testing day, the participants were provided with an ad libitum dinner and an evening snack packout, the latter of which was to be consumed at home throughout the evening before going to bed. A 7 day washout period occurred between breakfast patterns.

### 2.2. Study Participants

Between August 2010 and May 2014, adolescent girls were recruited from the Columbia, MO, USA area through advertisements, flyers, and email list serves to participate in the study. In an effort to extend the findings from our previous study that recruited late adolescent teenage girls [7], a similar set of eligibility criteria was used with the additional recruitment of habitual breakfast consumers. Eligibility was determined through the following inclusion criteria: (1) age range of 13–20 years; (2) overweight to obese (body mass index: 25–34.9 kg/m^2^); (3) no metabolic or neurological diseases or other health complications; (4) not been clinically diagnosed with an eating disorder; (5) not currently or previously on a weight loss or other special diet in the past 6 months; (6) documented regular menstrual cycles between 21–36 days in duration for the past 6 months. In addition, a dietary questionnaire was completed to document weekly breakfast habits and/or specific foods consumed. The habitual breakfast skippers (SKIP) consumed breakfast ≤ 2 days/week, and the habitual breakfast consumers (CONSUME) ate a carbohydrate-rich breakfast (defined as a meal containing 80% of energy from carbohydrates) at least 5 days/week. 

A total of 350 teens conveyed interest in participating in the study. Fifty-six met the screening criteria, were available for the 8 h testing days, and began the study. Of these, 37 completed all study procedures (19 SKIP; 18 CONSUME). Of those who withdrew from the study, eleven withdrew due to non-compliance and eight withdrew because of scheduling issues. Demographic data of those who completed the study are shown in Table 1. 

Prior to signing the consent/assent forms, all potential participants were informed that the purpose of the study was to examine how the body responds to eating different types of breakfasts. They were blinded to the breakfast habits and breakfast protein quantity comparisons. However, all procedures and risks were discussed prior to signing the consent/assent forms. The study was approved by the MU Health Sciences institutional review board. The participants received a stipend of $150/testing day.

### 2.3. Breakfast Patterns

For 4 consecutive days/pattern, the participants were provided 350 kcal NP breakfasts (15% of energy as protein (13 g protein)/65% of energy as carbohydrates/20% of energy as fat) or HP breakfasts (40% of energy as protein (35 g protein)/40% of energy as carbohydrates/20% of energy as fat), in randomized order, to be consumed between 7–9:30 a.m. (prior to school). The caloric content of the breakfast meals was approximately 18% of total daily intake which was estimated by energy expenditure equations specific for adolescents [14]. The participants were blinded to which breakfast pattern they were receiving each week. Both patterns included ‘typical breakfast foods’ such as yogurt parfaits, bagels, breakfast burritos, cereals, etc. Since these were mixed meals, the participants were unable to determine which meals contained more or less protein. No differences in palatability, assessed using a 100 mm visual analog questionnaire, were observed between the NP and HP breakfasts (75 ± 4 vs. 74 ± 3 mm, *p* > 0.10). In order to assess adherence to the protocol, participants were asked to return the containers that the breakfasts were provided in, along with any uneaten food. Compliance was similar between the NP and HP treatments for all study participants, and was nearly 100%. 

### 2.4. Specific Testing Procedures on Day 4 of Each Pattern

The participants reported to the research facility between 6:00 a.m. and 9:00 a.m. after an overnight fast to complete the 8 h testing day. Each participant was seated in a reclining chair and, for the next 30 min, was acclimated to the room and became familiarized with the testing day procedures. A catheter was then inserted into the antecubital vein of the non-dominant arm and kept patent by saline drip throughout the remainder of the testing day. At time 15 min, a baseline (fasting) blood sample was drawn. At time 0 min, the respective breakfast and 8 oz water were provided during the NP and HP days. The participants consumed the breakfast within 30 min. Postprandial appetite and satiety questionnaires in combination with plasma blood samples were collected throughout the 8 h period. A standard lunch meal was provided 4 h post-breakfast during each testing day. The lunch was 500 kcal and contained 15% of energy as protein, 65% of energy as carbohydrates and 20% of energy as fat. At the end of the testing day, the catheter was removed and an ad libitum dinner and evening snack packout was provided to the participants as previously described [7]. 

### 2.5. Appetite and Satiety Questionnaires

Computerized questionnaires assessing appetite and satiety were completed prior to consuming breakfast (or the time scheduled to consume breakfast), and then every 30 min afterward until leaving the testing facility, using a computerized 100 mm visual analog scale. The previously validated questions were worded as “how strong is your feeling of hunger or fullness”, “how strong is your desire to eat”, and “how much food can you eat right now”, with anchors of “not at all” or “not much” to “extremely” or “an extreme amount” [15,16]. The scores on each question were used to calculate total net incremental area under the curve (AUC) for the perceived appetitive responses. The Adaptive Visual Analog Scale Software (Neurobehavioral Research Laboratory and Clinic; San Antonio, TX, USA) was used for these assessments.

### 2.6. Repeated Blood Sampling and Hormonal Analyses

Eighteen blood samples (4 mL/sample; 72 mL/testing day) were collected throughout each 8 h testing day. Specifically, blood was collected at −15, +0, +30, +45, +60, +90, +120, +150, +180, +210, +240, +270, +285, +300, +330, +360, +390, and +420 min. The samples were collected in test tubes containing ethylenediaminetetraacetic acid. Protease inhibitors (4-(2-Aminoethyl) benzenesulfonyl fluoride hydrochloride (CenterChem Inc; Norwalk, CT, USA) and dipeptidyl peptidase-4 inhibitor (Millipore; Burlington, MA, USA) were added to reduce protein degradation. Hydrogen chloride (FisherScientific; Hampton, NH, USA) was added based on the ghrelin assay. Within 10 min of collection, the samples were centrifuged at 4 °C for 10 min. The plasma was separated and stored in microcentrifuge tubes at 80 °C for future analysis. Plasma active ghrelin and total peptide YY (PYY) were measured using the Milliplex MAP magnetic bead-based multianalyte, a metabolic panel, 4 plex (Millipore; Burlington, MA, USA) and Magpix Luminex technologies (Luminex Corporation; Austin, TX, USA).

### 2.7. Data and Statistical Analyses

Total net area under the curve (AUC) for hunger, fullness, desire to eat, prospective food consumption, plasma PYY, and plasma ghrelin responses, measured throughout each 8 h testing day was determined. Since plasma PYY was not normally distributed, the data was transformed using a natural log function and reported as ln(PYY). Daily energy intake consumed throughout the day was calculated from the standardized breakfast and lunch intake in combination with the ad libitum dinner and snack packout. Boxplots were used to identify and eliminate hormonal outliers, defined as non-physiological concentrations >2SD above/below the mean. Following the elimination of outliers, per-protocol analysis was performed on the remaining individuals. 

Mixed factor analysis of variance approach was performed to compare the main effects of habitual breakfast group (SKIP vs. CONSUME), protein quantity of the breakfasts (NP meal vs. HP meal), and group × protein interactions for all study outcomes. Power calculations derived from a previous study conducted by our lab suggests a sample size of 18 is sufficient (80% power, α = 0.05) to detect differences in perceived appetite responses and circulating markers of satiety following the consumption of breakfasts varying in macronutrient composition [7]. 

Analyses were conducted using the statistical package for the social sciences (SPSS; version 21.0; Chicago, IL, USA). *p* < 0.05 was considered statistically significant. All data are reported at mean ± standard deviation (SD).

## 3. Results

### 3.1. Perceived Appetite Responses

**Figure 1:**
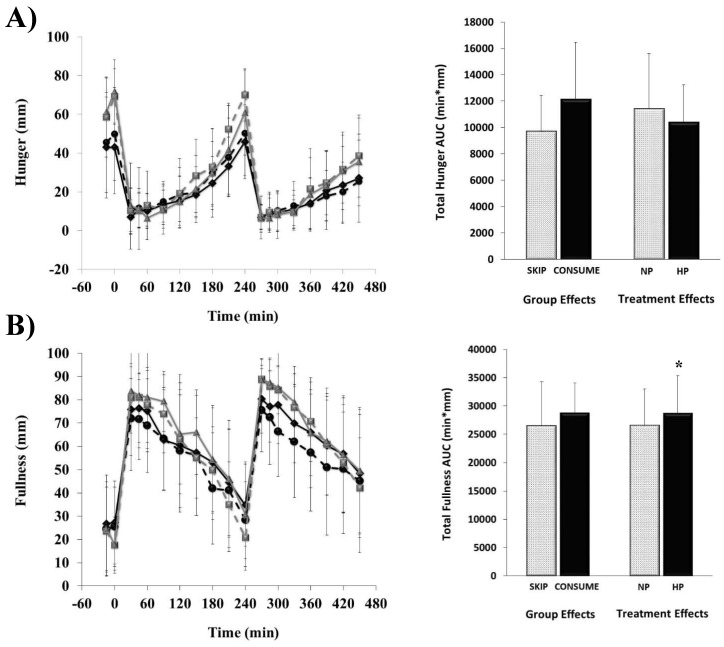
Perceived hunger (**A**) and fullness (**B**) responses throughout the testing days. The line graph displays the time course of change throughout the 8 h days. * denotes significance between breakfasts varying in protein quantity (*p* < 0.05). Breakfast was consumed at 0 min, and lunch was consumed at 240 min. Data are means ± SD. SKIP-NP (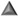), SKIP-HP (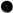), CONSUME-NP (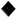), CONSUME-HP (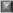).

**Figure 2:**
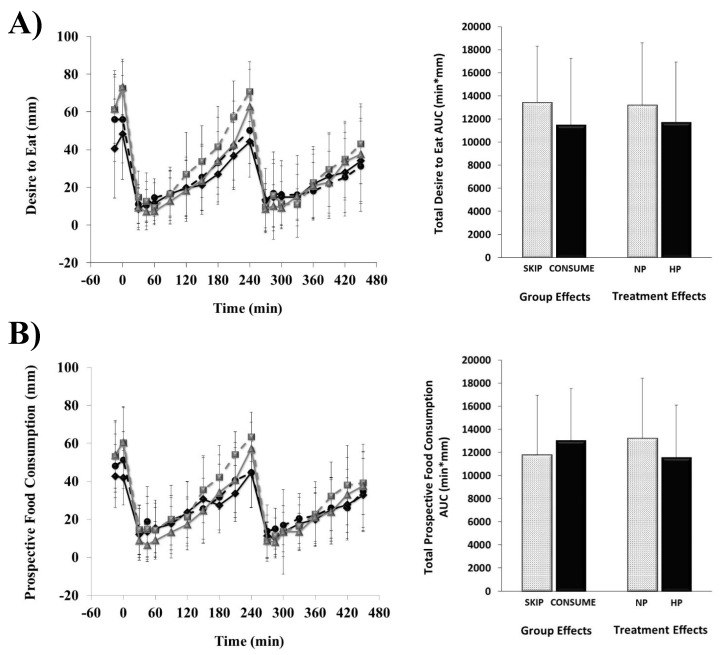
Perceived desire to eat (**A**) and prospective food consumption (**B**) responses throughout the testing days. The line graph displays the time course of change throughout the 8 h days. Breakfast was consumed at 0 min, and lunch was consumed at 240 min. Data are means ± SD. SKIP-NP (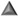), SKIP-HP (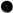), CONSUME-NP (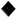), CONSUME-HP (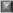).

Hunger, fullness, desire to eat, and prospective food consumption responses completed every 30 min throughout each of the breakfast patterns (i.e., NP vs. HP) along with total AUC responses are shown in Figure 1 and Figure 2. 

No main effects of habitual breakfast group, protein quantity, or group × protein interactions were detected for hunger (all, *p* > 0.1), desire to eat (all, *p* > 0.05), or prospective food consumption (all, *p* > 0.1), respectively. Although no main effect of habitual breakfast group was detected for daily fullness (*p* = 0.19), the HP breakfast led to greater fullness throughout the testing day (29,030 ± 6010 min × mm) compared to the NP breakfast (26,910 ± 5580 min × mm; *p* = 0.03).

### 3.2. Hormonal Responses

**Figure 3:**
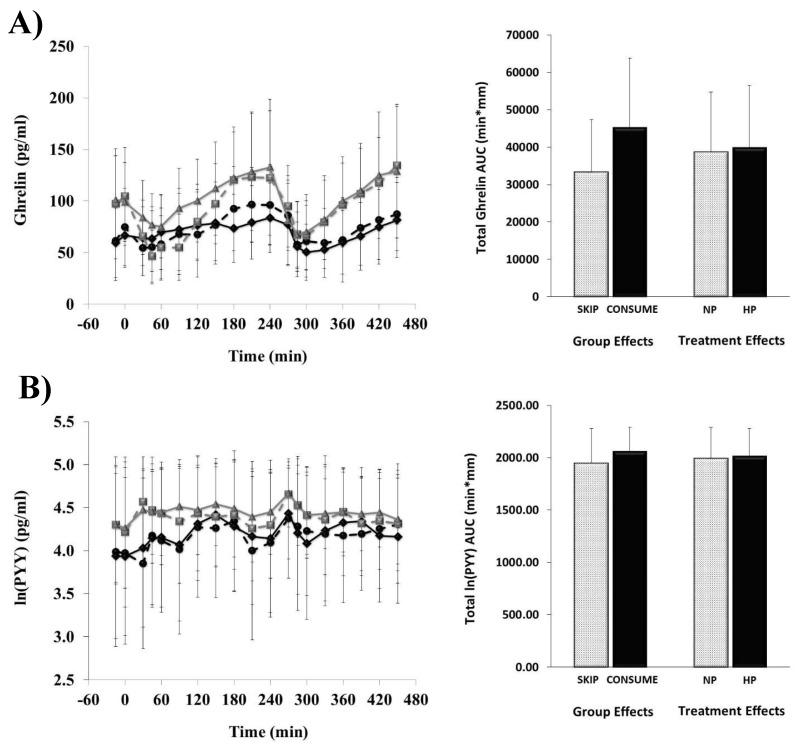
Circulating ghrelin (**A**) and PYY (**B**) throughout the testing days. The line graph displays the time course of change throughout the 8 h days. Breakfast was consumed at 0 min, and lunch was consumed at 240 min. Data are means ± SD. SKIP-NP (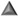), SKIP-HP (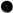), CONSUME-NP (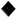), CONSUME-HP (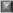).

The ghrelin and ln(PYY) responses completed every 30 min throughout each of the breakfast patterns (i.e., NP vs. HP) along with total AUC responses are shown in Figure 3.

No main effects of habitual breakfast group, protein quantity, or group × protein interactions were detected for ghrelin (all, *p* > 0.1) and ln(PYY) AUC (all, *p* > 0.1).

### 3.3. Energy Intake Responses

Although daily energy was not different between groups or within breakfasts varying in protein quantity, daily protein consumption was greater, and daily carbohydrate consumption was lower following the HP vs. NP breakfast patterns (Table 2). In addition, a group × protein interaction was detected for daily fat consumption (*p* < 0.01) such that the consumption of the HP breakfast led to lower daily fat intake vs. NP breakfast within the SKIP participants (*p* < 0.01). This effect was not observed within the CONSUME group. 

## 4. Discussion

We sought to examine the effects of habitual breakfast patterns and breakfast quality on postprandial appetite, satiety, and food intake in overweight adolescent girls. Regardless of habitual breakfast patterns, the consumption of a HP breakfast increased daily fullness and protein intake while reducing total carbohydrate consumption compared to the NP breakfast. Although habitual breakfast patterns had no direct effect on the study outcomes, the reduction in daily fat intake following the HP breakfast pattern was only observed within the habitual SKIP participants. These data suggest that the recommendation to consume a HP breakfast for improved satiety and ingestive behavior is appropriate for overweight adolescents girls, regardless of habitual breakfast patterns. 

The universal belief that breakfast is beneficial for health outcomes has recently come under scrutiny due to the limited and inconclusive experimental evidence [17]. Although breakfast type and size are critical factors for consideration [5], habitual breakfast patterns also influence the appetitive and/or metabolic response to breakfast (skipping) [12]. Alwattar et al. reported differences in glucose concentrations that were dependent on habitual breakfast consumption. When compared to those who habitually consumed breakfast, the habitual breakfast skippers experienced greater glucose concentrations throughout the day following the consumption of a HP breakfast vs. NP breakfast [8]. Additionally, Thomas et al. showed that habitual breakfast consumers elicited greater hunger responses and reduced satiety following breakfast consumption compared to habitual breakfast skippers, regardless whether breakfast was consumed or skipped [12]. Previous evidence from another group suggests that ghrelin concentrations are influenced by habitual meal timing [18,19]. Thus, although we expected to observe lower circulating ghrelin within the SKIP vs. CONSUME group, pre- and postprandial ghrelin responses were not different. It is possible that the study acclimation days, particularly within the breakfast skipping group, negated any differences in habitual ghrelin responses [18,19]. These findings suggest that the ghrelin response to a dietary intervention, like breakfast, occurs transiently. Furthermore, the transient nature of ghrelin also reflects the lack of differences in measures of perceived appetite (i.e., hunger, fullness, desire to eat, and prospective food consumption) such that any other hypothesized differences are quickly negated by the implementation of acclimation days. It is unclear whether the lack of differences in these measures of perceived appetite responses are a result of mitigated differences in ghrelin or whether the differences wane simultaneously with ghrelin. 

Acute feeding trials consistently illustrate increased satiety, as evidenced from postprandial and daily increases in fullness, following the consumption of higher-protein breakfasts, containing 25–30 g protein, compared to the average American breakfast that contains ~15 g protein [20,21]. Plasma PYY has been proposed as the gastrointestinal satiety mechanism since some, but not all studies, illustrate increased PYY concentrations with protein consumption [7]. Although the fullness data within the current study is in agreement with the current body of literature, plasma PYY concentrations in this study were not different. One explanation may be due to differences in protein source. Although both breakfast treatments were mixed meals with a combination of plant and animal proteins, the higher-protein meals contained predominately beef and egg protein and the normal-protein breakfast meals contained primarily dairy and wheat protein. However, in studies that measure subjective appetite responses in combination with hormonal concentrations, only about 40% detect increases in fullness and increases in circulating PYY following the consumption of higher-protein vs. normal-protein meals [22]. Thus, the lack of differences in the current study are not atypical. Traditionally, PYY has been proposed as a potent regulator of satiety [23]. This traditional role has since been challenged by a recent review, suggesting physiological differences do not necessarily mediate meaningful differences in satiety [24]. The findings in this study support the latter view. Future research should continue to elucidate the role of this hormone in regulating perceived satiety. 

A previous acute crossover trial from our lab observed decreased activation in reward driven eating via reduced activation in reward regions of the brain and decreased food cravings following the consumption of breakfasts varying in macronutrient composition [7,25]. These responses were also associated with reductions in evening snacking of unhealthy snack foods [7]. The findings from the current study are consistent with the aforementioned differences in food choice. However, it is possible that the increased protein consumption in combination with reduced carbohydrates was a result of the breakfast composition within the HP vs. NP meals. Thus, it is unclear whether these differences are driven by the breakfast alone or changes in evening snacking.

The limitations of this study are inherent within the nature of all acute crossover design studies. First, current controversy exists as to whether the acute satiety response following a single HP breakfast translates to reductions in subsequent meal and/or daily food intake. Although some acute studies detect a predictive relationship between perceived appetite and subsequent food intake [16,26], this relationship is inconsistent [27,28]. Thus, it is unclear whether changes in perceived daily fullness will alter subsequent meal and/or daily food intake. In addition, only girls were included in the current study. Thus, future studies are necessary to determine whether these findings also extrapolate to overweight adolescent boys. Finally, although PYY is highly correlated with protein intake [22], other anorexigenic hormones (i.e., GLP-1, leptin, etc.) are secreted in response to protein consumption/protein diets and might facilitate increased fullness. However, these hormones were not examined within the context of this study. 

## 5. Conclusions

In summary, habitual breakfast patterns do not influence the majority of appetitive, hormonal, and ingestive behavior responses to HP vs. NP breakfasts. However, the consumption of a HP breakfast increased daily fullness, increased daily protein consumption, and reduced daily carbohydrate consumption compared to the NP breakfast but did not influence total caloric intake over 24 h. Collectively, these data suggest that the recommendation to consume a HP breakfast for improved satiety and ingestive behavior is appropriate for overweight adolescent girls, regardless of habitual breakfast patterns.

## Figures and Tables

**Table 1:** Participant characteristics for habitual breakfast skippers (SKIP) and consumers (CONSUME). Data are reported as means ± SD (when applicable).

	SKIP (*n* = 19)	CONSUME (*n* = 18)
Age (year)	19 ± 1	19 ± 1
Height (cm)	166 ± 5.4	167 ± 7.0
Weight (kg)	80.3 ± 9.9	80.3 ± 9.9
BMI (kg/m^2^)	29.0 ± 3.8	28.9 ± 2.9
Frequency of breakfast consumption (no/week)	1 ± 1	6 ± 1
First eating or drinking occasion of the day	12:30 ± 0:15 p.m.	8:15 ± 0:10 a.m.

**Table 2:** Daily energy content and macronutrient intake following the consumption of a normal-protein breakfast (NP) and a higher-protein breakfast (HP) in habitual breakfast skippers (SKIP) and consumers (CONSUME). Data are reported as means ± SD.

	SKIP		CONSUME		Group Effect	Protein Effect	Group × Protein
	NP	HP	NP	HP			
Energy (kcal)	2360 ± 138	2312 ± 132	2554 ± 145	2542 ± 138	0.49	0.22	0.29
Protein (g)	76 ± 4	95 ± 3	79 ± 4	101 ± 3	0.92	<0.01	0.15
Carbohydrates (g)	346 ± 20	325 ± 21	366 ± 21	343 ± 22	0.71	<0.01	0.14
Fat (g)	65 ± 5	61 ± 5	79 ± 6	79 ± 6	0.12	<0.01	<0.01
